# Supplementary material for: EryDB: A Transcriptomic Profile Database for Erythropoiesis and Erythroid-related Diseases
Source: Genomics Proteomics Bioinformatics. 2024 Apr 2;23(2):qzae029. doi: 10.1093/gpbjnl/qzae029 (PMC13223742; doi:10.1093/gpbjnl/qzae029)
Supplement: qzae029_Supplementary_Data [file qzae029_supplementary_data.zip › Table S2-done.docx]

| **Table S2 Content of the datasets that can be searched by users** | | | |
| --- | --- | --- | --- |
| **Item** | **Amount** | **Content** |  |
| Cell types | 12 | HSC, MPP, CMP, MEP, BFU-E, CFU-E, Pro-E, Baso-E, Poly-E, Ortho-E, Retic, and RBC |  |
| Disease types | 5 | Fanconi anemia, Diamond-Blackfan anemia, hemoglobinopathies (including sickle cell disease and thalassemia), aplastic anemia, and epo-resistant anemia |  |
| Compound types | 9 | TRβ agonists, ERBB inhibitors, IDH2 mutant-specific inhibitors, PPAR-α agonists, HbF regulators, BET bromodomain inhibitors, erythropoietin, iron, and corticosteroids |  |
| Species | 3 | *Homo sapiens*, *Mus musculus*, and *Danio rerio* |  |
| Tissue types | 11 | Bone marrow, cord blood, peripheral blood, spleen, embryo, fetal tissue, heart, kidney, iPSC, cell line, and other (unknown). |  |
| Experimental types | 2 | *In vivo* and *in vitro* |  |
| Omics techniques | 2 | Bulk RNA-seq and scRNA-seq |  |

*Note*: HSC, hematopoietic stem cell; MPP, multipotent progenitor cell; CMP, common myeloid progenitor; MEP, megakaryocyte-erythroid progenitor; BFU-E, burst-forming unit-erythroid; CFU-E, colony-forming unit-erythroid; Pro-E, proerythroblast; Baso-E, basophilic erythroblast; Poly-E, polychromatophilic erythroblast; Ortho-E, orthochromatic erythroblast; Retic, reticulocyte; RBC, red blood cell; BET, bromodomain and extra-terminal domain; TRβ, thyroid hormone receptor β; ERBB, epidermal growth factor receptor; IDH2, isocitrate dehydrogenase 2; PPAR-α, peroxisome proliferator-activated receptor α; HbF, fetal hemoglobin; iPSC, induced pluripotent stem cells.
